# Instruments for assessing organisational food environments of workplaces: a scoping review

**DOI:** 10.1017/S1368980025000576

**Published:** 2025-04-14

**Authors:** Ana Beatriz Coelho de Azevedo, Patricia Gálvez Espinoza, Fernanda Martins de Albuquerque, Cintia Chaves Curioni, Daniel Henrique Bandoni, Daniela Silva Canella

**Affiliations:** 1 Postgraduate Program in Food, Nutrition and Health, Rio de Janeiro State University, Rio de Janeiro, Brazil; 2 Department of Nutrition, Chile University, Santiago, Chile; 3 Department of Social Nutrition, Rio de Janeiro State University, Rio de Janeiro, Brazil; 4 Center of Practices and Research in Nutrition and Collective Food Services, Federal University of São Paulo, Santos, Brazil; 5 Department of Applied Nutrition, Rio de Janeiro State University, Rio de Janeiro, Brazil

**Keywords:** Organisational food environment, Workplaces, Scoping review, Instruments

## Abstract

**Objective:**

To map out evidence on instruments for evaluating organisational food environments of workplaces and the components and dimensions considered in the identified instruments.

**Design:**

A scoping review that includes studies published as of January 2005, the year of publication of the model developed by Glanz *et al.* (2005). The databases consulted were PubMed, Embase, Web of Science, PsycINFO, Scopus and Google Scholar until November 2024, without language restrictions. Studies were included if they evaluated the food environment of workplaces such as companies/factories, universities/post-secondary institutions/technical colleges and hospitals/health care units. The conceptual model of Castro and Canella (2022), considering its components and dimensions, was used to synthesise the data.

**Results:**

After a full reading, fifty-four articles were selected. Most were conducted in the United States and Brazil, although there were studies from sixteen countries. A total of thirty-six instruments were identified: nineteen were used in universities, eight in hospitals, and eleven in companies. No instrument included all components and dimensions of the conceptual model; however, three instruments included most of them. The most evaluated component was the internal level of eating spaces, and the most evaluated dimensions were the availability and quality of foods/beverages in eating spaces. Of the thirty-six instruments, twenty-nine reported some measure of validity or reproducibility. The limitation most reported by the studies was the non-generaliation of results because samples are limited.

**Conclusions:**

Evaluations of the organisational food environment of workplaces can be used for monitoring, planning interventions and formulating public policies for such places, thereby enhancing workers’ health.

The overall prevalence of obesity among adults has been increasing alarmingly in recent decades. A study with data from 196 countries showed that such prevalence more than doubled between 1990 and 2022^(1)^. According to the World Obesity Federation, the prevalence of adults with excess weight (overweight or obesity) in 2020 was 42 %, with an estimated increase to 54 % in 2035, which is equivalent to 1·77 billion adults with overweight and 1·53 billion with obesity^(2)^.

Workplaces, where many adults spend much of the day, play a crucial role in this scenario. More than one-third of the global workforce regularly works more than 48 h a week, which can affect the balance between personal and professional life, and negatively impact the health and well-being of workers^(3,4)^. In this sense, workplaces can be a barrier or a facilitator to proper and healthy eating.

This is the context of the debate about the importance of the organisational food environment, defined as ‘the place where food is sold or supplied to workers, students or other members of institutions and organisations. It includes schools, universities, businesses, public services, hospitals, prisons and civil society organisations, as well as their respective food venues (snack bars, kiosks and food vending machines)’^(5)^. The conceptual model proposed by Castro and Canella advanced in the systematisation and definition of the components and dimensions that compose the organisational food environment, showing the complexity of this environment, seeking to overcome the limitations of the approach usually adopted by them, which is restricted to the availability of food in the eating spaces of organisations^(6,7)^.

The model considers four components: the institutional level (elements of the physical environment existing in the organisation that influence food choices and practices), the internal level of eating spaces (the elements of the food environment within each commercial and non-commercial eating space), the decision level (the governance of the organisation’s food environment, power relations, and decision-making, which occurs in the external and internal spheres of the organisation) and surroundings (the physical and virtual contexts related to foods that are available and are not interfered with by the management of the organisation)^(6)^. In addition, to shed light on the complexity of the institutional level and the internal level of eating spaces, the model also includes ten dimensions related to these levels: availability, accessibility, affordability, quality, food and nutrition information, promotion of food, beverages and culinary preparations; acceptability, convenience, ambience and infrastructure of eating spaces^(6)^.

Organisational food environments can play a strategic role in improving workers’ health, and both can be improved by evaluating such environments properly to advance more effective public policies and interventions. However, there seem to be no instruments that cover all the important elements for measuring these environments. Furthermore, there is no specific review of the literature on organisational food environments. According to available reviews on food environment evaluation, most of the instruments found in the literature were developed in the United States; few were validated, and the different measures, definitions and approaches being used make it difficult to compare the studies. In addition, the reviews identified few instruments available to evaluate workplaces, and there was no clear definition of what a workplace is, which may have limited the identification of instruments^(7,8)^.

In this context, this study aimed to map out evidence on instruments for the evaluation of organisational food environments of workplaces, as well as to identify the components and dimensions considered in them.

## Methods

A scoping review was performed to systematise evidence on instruments for evaluation of the organisational food environment. The protocol associated with this review – including the databases and search terms – was published by BMJ Open^(9)^.

The search was carried out in the following databases: PubMed, Embase, Web of Science, PsycINFO, Scopus and Google Scholar. It included studies published in peer-reviewed journals as of January 2005, which is the year of publication of the conceptual model of a healthy nutrition environment developed by Glanz and collaborators (2005), an important starting point for studies of food environment measurement^(10)^. To allow the selection of a wide range of articles, no language restriction was applied. Searches were completed on February 20^th^, 2024 and updated on November 6^th^, 2024.

These are the research questions in this review: ‘What instruments are available to evaluate the organisational food environments of workers?’; ‘Which workplaces have been studied?’; ‘What elements of organisational food environments have been studied in different types of environments?’; ‘Have the psychometric properties of instruments and indicators been evaluated?’ The search terms were defined based on previous studies, bibliographical research on the subject and the experience of the researchers. The search strategy was adapted for each database. More details about the search terms can be found in the review protocol^(9)^.

The search considered not only methodological studies but also studies whose objective was to evaluate the organisational food environment. Eligible studies were selected according to the Population–Concept–Context structure, recommended by the Joanna Briggs Institute^(11)^. The population consisted of workplaces (companies/industries – henceforth referred to only as companies, universities/post-secondary institution/technical college, referred as universities and hospitals/health care units – referred to only as hospitals), components (institutional level; internal level of eating spaces; decision level and surroundings) and eating spaces evaluated in workplaces (such as commercial and non-commercial services and food vending machines^(6)^; for concept, the present review considered all studies that had evaluated at least one of the different dimensions of the organisational food environment according to Castro and Canella’s model (2022) (availability, accessibility, affordability, quality, food and nutrition information, and promotion of foods, beverages and culinary preparations; and availability, acceptability, convenience, ambience and infrastructure of eating spaces)^(12)^. As regards context, no geographical or population restrictions were applied.

The conceptual model proposed by Castro and Canella^(6)^ was used for data extraction and synthesis. Although other models contain the organisational food environment, their model is more comprehensive and looks further into the elements of such environment, thus outperforming the approach traditionally used for analysing these food environments, which is restricted to food availability in the eating spaces of organisations^(6,7)^.

The present study focused on workplaces such as companies, universities and hospitals because, despite the potential differences between these environments, they have many similarities; one of them is the public of adults/workers, who spend long hours in these places on a daily basis. Thus, these are the exclusion criteria: (1) studies that did not evaluate the organisational food environment; (2) studies that evaluated the food environment in schools, prisons and recreational facilities; (3) studies that evaluated hospitals from the perspective of patients and studies that evaluated universities from the perspective of students. Although schools, prisons and recreational facilities employ workers, the option not to include studies that evaluated them is mainly due to the priority public of these places and their specificities, such as young age, low autonomy of individuals (in the case of schools and prisons) and time of stay on site (very short stay in the case of recreational facilities and very long stay in prisons). All these aspects probably influence the characteristics of the environment. In addition, schools have so many specificities that particular models were developed for them^(12,13)^.

The search in electronic databases and the screening and removal of duplicates using the Rayyan online software^(14)^ was performed by a researcher. Then, two trained researchers independently reviewed and selected articles by title and abstract, excluding those not related to the topic of the review. First, the selection stage was pilot-tested with ten articles. After the agreement was confirmed, the researchers performed the review and selection of all articles. The full texts of the articles selected at this stage were evaluated for inclusion in the present review. Differences during the process were resolved by consensus among reviewers or by consulting a third reviewer. The selection process will be shown below in the PRISMA-SCR flow chart^(15)^.

For the selected articles, considering the inclusion and exclusion criteria, data extraction was performed using Google Forms^(16)^. A standardised data extraction form was developed and tested in the first ten articles selected previously and then refined. Two reviewers extracted the data independently, considering (1) reference, including the year of publication; (2) country; (3) sample; (4) study objectives; (5) study design; (6) environment (evaluated the organisational food environment of companies, universities or hospitals); (7) food environment components and eating spaces evaluated; (8) instruments and methodologies used for measuring the food environment; (9) measures dimensions of eating spaces^(6)^; (10) limitations and gaps pointed out by the authors and (11) reported validity and reliability of measures. As additional information, during the extraction, there was a need to check whether the study used objective measures, such as the evaluation of spaces or characteristics of the environment, or subjective measures, for example, the perceptions of workers or managers about certain characteristics of the environment.

The main results of interest in this study were the instruments used for evaluating the organisational food environment of workplaces and their components and dimensions.

First, the instruments were categorised by type of organisation (companies, universities and hospitals), to provide a comparison within types and between types. The main differences and similarities between the instruments were explored through a more detailed analysis. Additionally, when available, the reliability and validity measures used in the evaluation of the instruments were described, as well as the limitations pointed out by the authors of the studies.

## Results

**Figure 1.**
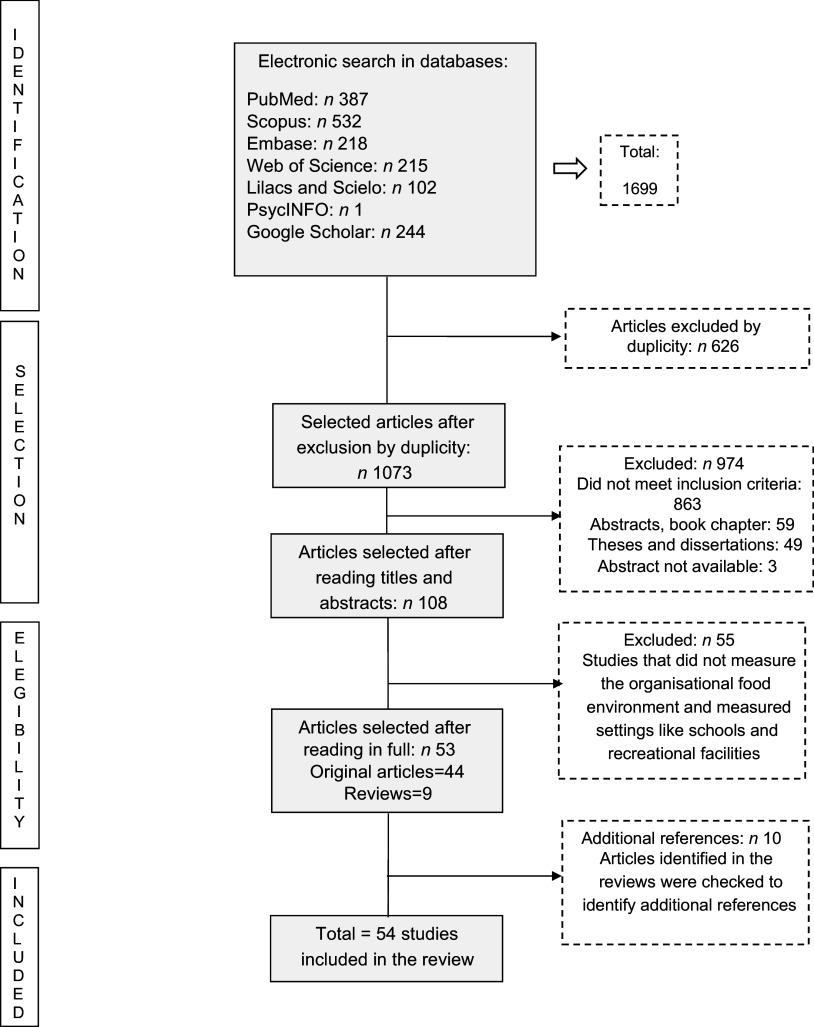
PRISMA flow diagram detailing the number of studies included and excluded at each stage of the screening process.

The initial search in the databases resulted in a total of 1699 studies. After the removal of the duplicates, 1073 articles remained, of which 108 were previously selected, based on their title and abstract. After the full texts were read, forty-four studies met the eligibility criteria. In addition to the original studies, nine reviews were identified and used for searching extra references. After consulting the reviews, ten additional original studies were identified, totalling fifty-four studies included in the present review. The main reason for exclusion was the lack of evaluation of the organisational food environment or the inclusion of places such as schools and recreational facilities. Studies were also excluded when they focused on university students and did not consider workers, as this study aims to evaluate workplaces. The selection process is presented in the PRISMA flow chart (Fig. 1).

### General characteristics of the studies

Of the fifty-four studies included, 46·3 % (*n* 25) were conducted in the United States and 14·8 % (*n* 8) in Brazil, while the others were carried out in other fourteen countries. Although the first study was published in 2007, several studies were published as of 2010. The highest number of articles (14·8 %; *n* 8) was published in 2022, followed by the years 2013 and 2021, both with five studies (9·3 %) (Table 1). Regarding study design, 57·4 % (*n* 31) were cross-sectional, 27·8 % (*n* 15) were methodological studies, 5·6 % (*n* 3) used mixed methods, 3·7 % (*n* 2) were ecological, 3·7 % (*n* 2) were cross-sectional and intervention studies and 1·9 % (*n* 1) had a quasi-experimental design (data not presented).

**Table 1:** Instruments identified for assessing the organisational food environment of companies, universities and hospitals, 2024

Study/Country	Instrument	Measure type	Components of the food environment				Measured dimensions of eating spaces										
			Institutional level	Internal level of eating spaces	Decisional level	Surroundings	Availability (institutional level)	Accessibility (institutional level)	Affordability	Quality	Food and nutrition information	Promotion	Availability of the eating space	Acceptability of the eating space	Convenience of the eating space	Ambiance of the eating space	Infrastructure of the eating space
Companies																	
Shimotsu *et al.*, 2007/USA^(17)^	WEM	Objective	✓	✓		✓	✓			✓		✓	✓				✓
Dejoy *et al.*, 2008/USA^(18)^	EAT	Objective	✓	✓	✓		✓		✓	✓	✓	✓	✓				
Beresford *et al.*, 2010/USA^(19)^	EAT	Objective	✓	✓			✓			✓		✓	✓				
Parker *et al.*, 2010/USA^(20)^	EAT	Objective	✓	✓	✓		✓		✓	✓	✓	✓	✓				
Apostolopoulos *et al.*, 2011/USA^(21)^	HEATWAI	Objective	✓	✓			✓			✓			✓				✓
Wong *et al.*, 2011/Mexico^(22)^	CHESS	Objective	✓	✓		✓	✓			✓	✓		✓				
Apostolopoulos *et al.*, 2012/USA^(23)^	HEATWAI	Objective	✓	✓			✓			✓			✓				✓
Hoehner *et al.*, 2013/USA^(24)^	WEBS	Objective/ Subjective	✓	✓		✓	✓	✓	✓			✓	✓				✓
Almeida *et al.*, 2014/USA^(25)^	CHEW	Objective	✓	✓			✓					✓					
Lillehoj *et al.*, 2015/USA^(26)^	NEMS-V	Objective	✓	✓			✓			✓			✓				
Charoenbut *et al.*, 2018/Thailand^(27)^	Questionnaire-based on CHEW and NEMS-S	Objective/subjective		✓	✓				✓	✓			✓	✓			
Banerjee, Reddy, Gavaravarapu, 2022/India^(28)^	Checklist	Objective/ Subjective	✓	✓			✓					✓	✓	✓		✓	✓
Shokeen, Tamber, Sinha, 2022/India^(29)^	CHEW	Objective	✓		✓	✓	✓					✓					
Quezada *et al.*, 2023/Chile^(30)^	Adaptation for Chile of the NEMS-S	Objective		✓		✓		✓	✓				✓				
Geboers *et al.*, 2024/Netherlands^(31)^	Survey aligned with the ANGELO framework’s definition of the food environment	Objective/subjective	✓	✓	✓		✓						✓	✓			✓
Universities																	
Freedman, 2010/USA^(32)^	U-NEAT	Objective	✓	✓					✓	✓	✓	✓	✓		✓		✓
Freedman, 2010/USA^(32)^	U-NPAT	Objective			✓												
Byrd-Bredbenner *et al.*, 2012/USA^(33)^	Assessment through inventory	Objective		✓						✓			✓				
Voss *et al.*, 2012/USA^(34)^	NEMS-V	Objective		✓						✓		✓	✓		✓		
Horacek *et al.*, 2013/USA^(35)^	NEMS-R	Objective	✓	✓		✓			✓	✓	✓	✓	✓				
Horacek *et al.*, 2013/USA^(35)^	NEMS-CD	Objective	✓	✓		✓			✓	✓	✓	✓	✓				
Horacek *et al.*, 2013/USA^(36)^	Adaptation of NEMS-S	Objective		✓		✓			✓	✓			✓				
Lo *et al.*, 2016/Canada^(37)^	NEMS-GG	Objective	✓	✓		✓	✓		✓	✓	✓		✓		✓		
Roy *et al.*, 2016/Australia^(38)^	The food environment-quality index	Objective	✓	✓			✓	✓		✓		✓	✓				
Tseng *et al.*, 2016/USA^(39)^	NEMS-CD	Objective	✓	✓		✓			✓	✓	✓	✓	✓				
Tseng *et al.*, 2016/USA^(39)^	NEMS-S	Objective	✓	✓		✓			✓	✓			✓				
Pulz *et al.*, 2017/Brazil^(40)^	NEMS-R	Objective	✓	✓			✓	✓	✓	✓	✓	✓			✓		
Horacek *et al.*, 2018/USA^(41)^	A modified version of the NEMS-S	Objective		✓		✓		✓	✓	✓			✓				
Bortolot, Perez, Franco, 2019/Brazil^(42)^	Instrument for analysing the university food environment	Objective	✓	✓			✓	✓	✓	✓		✓	✓				
Horacek *et al.*, 2019/USA^(43)^	NEMS-V	Objective		✓						✓		✓	✓		✓		
Horacek *et al.*, 2019/USA^(43)^	(VEND)ing audit	Objective	✓	✓			✓	✓	✓	✓	✓	✓	✓		✓		
Roy *et al.*, 2019/New Zealand^(44)^	The food environment-quality index	Objective	✓	✓			✓	✓	✓			✓	✓	✓			
Roy *et al.*, 2019/New Zealand^(44)^	Survey with questions about food purchasing behaviours, determinants and opinions	Subjective	✓	✓			✓		✓	✓	✓		✓	✓	✓		✓
Lee *et al.*, 2020/Canada^(45)^	NEMS-UC	Objective		✓					✓		✓	✓	✓		✓		
Mann *et al.*, 2021/Australia^(46)^ Keat, Dharmayani, Mihrshahi, 2024/Australia^(47)^	The Uni-Food tool	Objective	✓	✓	✓		✓	✓	✓	✓	✓	✓	✓		✓		
Martinez-Perez, Arroyo-Izaga, 2021/Spain^(48)^	Assessment through inventory	Objective	✓	✓			✓		✓	✓		✓	✓				
Rodrigues *et al.*, 2021/Brazil^(49)^	Questionnaire	Objective	✓	✓			✓		✓	✓	✓	✓	✓		✓		
Tavares *et al.*, 2021/Brazil^(50)^	Instrument for analysing the university food environment	Objective	✓	✓			✓		✓		✓	✓	✓		✓		
Franco *et al.*, 2022/Brazil^(51)^	Instrument for analysing the university food environment	Objective	✓	✓			✓		✓		✓	✓	✓		✓		✓
Han *et al.*, 2022/China^(52)^	NESC-CC	Objective	✓	✓			✓			✓		✓	✓				✓
Kochergina *et al.*, 2022/Russia^(53)^	Checklist based on NEMS	Objective		✓									✓	✓	✓		✓
Martinez-Perez *et al.*, 2022^(54)^	Survey on food purchasing, food choice behaviours and opinions	Subjective		✓					✓	✓			✓	✓	✓		
Mensah *et al.*, 2022/Gana^(55)^	Survey instrument developed based on NEMS-R and NEMS-S	Objective		✓		✓	✓	✓		✓							
Batista *et al.*, 2023/Brazil^(56)^	Instrument for analysing the university food environment	Objective	✓	✓			✓		✓		✓	✓	✓		✓		
Rodrigues *et al.*, 2023/Brazil^(57)^	Questionnaire	Objective		✓							✓	✓	✓		✓		
Li *et al.*, 2024/China^(58)^	C-NEMS-S	Objective	✓	✓			✓	✓	✓	✓			✓		✓		
Hospitals																	
Lawrence *et al.*, 2009/USA^(59)^	Assessment tool for vending machines	Objective		✓	✓					✓		✓	✓		✓		
Lesser *et al.*, 2012/USA^(60)^	NEMS-C	Objective	✓	✓			✓		✓	✓	✓	✓	✓				
Winston *et al.*, 2013/USA^(61)^	HNES	Objective		✓						✓	✓	✓	✓				
Winston *et al.*, 2013/USA^(62)^	HNES	Objective		✓					✓		✓	✓	✓				
Lederer *et al.*, 2014^(63)^	Survey tool	Subjective			✓					✓							
Derrick, Bellini, Spelman, 2015/USA^(64)^	HNES	Objective		✓							✓	✓	✓				
Sharma *et al.*, 2016/USA^(65)^	EAT	Objective	✓	✓	✓		✓		✓	✓	✓	✓	✓				
James *et al.*, 2017/United Kingdom^(66)^	C-NET	Objective	✓	✓			✓			✓	✓	✓	✓				
Tsai *et al.*, 2018/Australia^(67)^	Food Environment Audit Tool	Objective	✓	✓			✓			✓		✓	✓				
José, Castro, Canella, 2021/Brazil^(68)^	Assessment Instrument of the Hospital Food Environment	Objective/subjective	✓	✓	✓	✓	✓	✓	✓	✓	✓	✓	✓		✓	✓	✓
Horton *et al.*, 2022/USA^(69)^	HNES	Objective	✓	✓			✓	✓	✓	✓		✓	✓				
Wierda *et al.*, 2024/Netherland^(70)^	Checklist inspired on the HNES	Objective	✓		✓		✓		✓	✓			✓				✓

CHESS, Community Health Environmental Scan Survey; CHEW, Checklist for Health Promotion Environments at Worksites; C-NEMS-S, Chinese Version of the Nutrition Environment Measurement Tool for Stores; C-NET, Consumer Nutrition Environment Tool; EAT, Environmental Assessment Tool; HEATWAI, Healthy Trucking Work-settings Audit Instrument; HNES, Hospital Nutrition Environment Scan for Cafeterias, Vending Machines, and Gift Shops; NEMS-C, Nutrition Environment Measures Survey for cafeteria; NEMS-CD, Nutrition Environment Measures Survey for campus dining; NEMS-GG, Nutrition Environment Measures Survey Grab-and-Go tool; NEMS-R, Nutrition Environment Measures Survey for Restaurants; NEMS-S, Nutrition Environment Measures Survey for stores; NEMS-UC, Nutrition Environment Measures Survey for University Campuses; NEMS-V, Nutrition Environment Measures Survey Vending; NESC-CC, Nutrition Environment Scoring for Chinese Style University/Work-site Canteens; U-NEAT, The University Nutrition Environment Assessment Tool; U-NPAT, The University Nutrition Program Assessment Tool; (VEND)ing audit, Vending Evaluation for Nutrient-Density; WEBS, Worksite and Energy Balance Survey; WEM, worksite environment measure.

### Instruments used

Table 1 shows the description of the identified instruments. In total, thirty-six instruments were used in universities (19), companies (11) and hospitals (8). Two of the instruments used in companies were also used in universities (NEMS-V) and hospitals (Environmental Assessment Tool). Of the identified instruments, seven were variations of the Nutrition Environment Measures Survey (NEMS), four were adaptations of NEMS versions, and twenty-five were original instruments. The most widely used instrument was the Nutrition Environment Measures Survey for Stores (NEMS-S). It was used in five studies and adapted in four of them. Other widely used instruments were the Environmental Assessment Tool, the Hospital Nutrition Environment Scan for Cafeterias, Vending Machines and Gift Shops and the Instrument for Analysis of the University Food Environment, each used in four studies (Table 1).

### Methodologies identified

Most studies used objective measurements. However, six studies used objective and subjective measures^(24,27,28,31,38,68)^, and two used only subjective measures^(54,63)^ to evaluate the perception of workers and managers about certain variables of interest. In addition, two studies carried out the evaluation using inventory analysis but without standardised instruments^(33,48)^ (Table 1).

### Dimensions evaluated

The most evaluated dimensions were availability and quality of foods/beverages in eating spaces, present in 88·9 % (*n* 32) and 72·2 % (*n* 26) of the instruments, respectively. The availability of establishments, promotion and affordability were evaluated in 63·9 % (*n* 23), 61·1 % (*n* 22) and 58·3 % (*n* 21) of the instruments, respectively. Although 25·9 % (*n* 14) of the included studies considered the surroundings, only three instruments^(22,24,68)^ included the surroundings as a formal component; in the others, they were evaluated separately. No instrument covered all components and dimensions of the target model. However, some are more complete, such as the Assessment Instrument of the Hospital Food Environment^(68)^, which evaluates all components and dimensions except for acceptability, and The Uni-Food Tool^(46,47)^, which does not evaluate the surroundings, acceptability, ambience and infrastructure of eating spaces, and the (VEND)ing audit^(43)^, which does not evaluate the decision level, surroundings, acceptability, ambience and infrastructure of eating spaces (Table 1).

### Evaluated workplaces

#### Companies

Of the eleven instruments used in companies, nine were original instruments^(17–25,27–29,31)^ developed specifically for this context, while two were not developed specifically for companies: the Nutrition Environment Measures Survey-Vending (NEMS-V)^(26)^ and an adaptation of the Nutrition Environment Measures Survey for Stores (NEMS-S) for Chile^(30)^. All instruments evaluated the internal level of eating spaces, while 81·8 % (*n* 9) evaluated the institutional level, and 27·3 % (*n* 3) considered the decision level. The surroundings were evaluated by 33·3 % of the studies (*n* 5); however, only two instruments^(22,24)^ included the surroundings as a formal component. The most evaluated dimensions were the availability of food and beverages in eating spaces (90·9 %; *n* 10) and the availability of establishments (81·8 %; *n* 9). Other dimensions frequently evaluated were quality (54·5 %; *n* 6), promotion (45·5 %; *n* 5), infrastructure (45·5 %; *n* 5) and affordability (36·4 %; *n* 4). No study evaluated the convenience of eating spaces (Table 1).

#### Hospitals

Eight instruments were used in studies that evaluated hospitals. The Hospital Nutrition Environment Scan for Cafeterias, Vending Machines and Gift Shops was the most frequent; it was used in four studies^(61,62,64,69)^, and one of the studies used NEMS for Cafeterias (NEMS-C)^(60)^. Among the instruments, 87·5 % (*n* 7) evaluated the internal level of eating spaces and 75 % (*n* 6) evaluated the institutional level. The decision level was evaluated by 50 % (*n* 4) of the instruments. Only one instrument/study evaluated the surroundings^(68)^. The most evaluated dimensions were the availability of food and beverages in eating spaces (100 %; *n* 8), promotion (87·5 %; *n* 7), quality (87·5 %; *n* 7), the availability of establishments (75 %; *n* 6) and affordability and food and nutrition information (62·5 %; *n* 5). No instrument evaluated the acceptability of eating spaces (Table 1).

#### Universities

In studies that evaluated universities, nineteen instruments were identified; six of them were variations of NEMS (NEMS-Campus Dining, NEMS-Grab and Go, NEMS-S, NEMS-Restaurants, NEMS-V and NEMS-University Campuses)^(34–37,39,40,43,45)^ and three instruments were adaptations of NEMS-S^(36,41,58)^. The most used instrument was developed by Franco *et al.*, and it was used in four studies^(37,39,40,45)^. The internal level of eating spaces was evaluated by 94·7 % (*n* 18) of the instruments, while the institutional level was evaluated by 63·2 % (*n* 12). The surroundings were evaluated by 29·6 % (*n* 8) of the studies and only 10·5 % (*n* 2) of the instruments evaluated the decision level. The most evaluated dimensions were the availability of foods and beverages in eating spaces (84·2 %; *n* 16) and quality (78·9 %; *n* 15). In addition, affordability was evaluated by 68·4 % (*n* 13), while promotion and convenience were evaluated by 57·9 % (*n* 11) of the instruments. No instrument evaluated ambience (Table 1).

### Reported validity and reproducibility

Of the thirty-six instruments, 72·2 % (*n* 26) reported some measure of validity, and 33·3 % (*n* 12) reported some measure of reproducibility. Of these, three were used in hospitals, nine in companies and sixteen in universities. Two instruments were used in more than one place; NEMS-V was used in universities and companies, and EAT was used in companies and hospitals. The most common measure of reproducibility was the agreement between evaluators (inter-rater reliability), which was used by 55·6 % (*n* 20) of the instruments, followed by the test–retest and used by 22·2 % (*n* 8) of the instruments. The most used validity measures were face validity (22·2 %, *n* 8) and content validity (16·7 %, *n* 6). All studies reported satisfactory results of the measurements performed (Table 2).

**Table 2:** Summary of instruments assessing the organisational food environment reporting validity and reliability of measures, 2024

Reference	Instrument	Setting	Reliability	Validity
Shimotsu *et al.*, 2007^(17)^	WEM	Company	Inter-rater	–
Dejoy *et al.*, 2008;^(18)^ Beresford *et al.*, 2010;^(19)^ Parker *et al.*, 2010;^(20)^ Sharma *et al.*, 2016^(65)^	EAT	Company /Hospital	Inter-rater	Face and concurrent validity
Freedman, 2010^(32)^	U-NEAT U-NPAT	University	–	Construct, content and face validity
Apostolopoulos *et al.*, 2011;^(21)^ Apostolopoulos *et al.*, 2012^(23)^	HEATWAI	Company	Inter-rater	–
Wong *et al.*, 2011^(22)^	CHESS	Company	Inter-rater	–
Lesser *et al.*, 2012^(60)^	NEMS-C	Hospital	Inter-rater	Face validity
Voss *et al.*, 2012;^(34)^ Horacek *et al.*, 2019^(43)^	NEMS-V	University	Inter-rater, test–retest	–
Winston *et al.*, 2013;^(61)^ Winston *et al.*, 2013;^(62)^ Derrick, Bellini, Spelman, 2015;^(64)^ Horton *et al.*, 2022^(69)^	HNES	Hospital	Inter-rater, intraclass	Content and face validity
Hoehner *et al.*, 2013^(24)^	WEBS	Company	Test–retest, internal consistency	–
Horacek *et al.*, 2013;^(35)^ Pulz *et al.*, 2017^(40)^	NEMS-R	University/ Post-secondary institution/Technical college	Inter-rater, test–retest	–
Horacek *et al.*, 2013;^(35)^ Tseng *et al.*, 2016^(39)^	NEMS-CD	University/ Post-secondary institution	Inter-rater, test–retest, intraclass	Face validity
Horacek *et al.*, 2013;^(36)^ Tseng *et al.*, 2016^(39)^	NEMS-S	University/Technical college	Inter-rater, test–retest, intraclass	–
Almeida *et al.*, 2014^(25)^	CHEW	Company	Inter-rater	–
Lillehoj *et al.*, 2015^(26)^	NEMS-V	Company	Inter-rater, test–retest	–
Lo *et al.*, 2016^(37)^	NEMS-GG	University	Inter-rater	Face and construct validity
Roy *et al.*, 2016;^(38)^ Roy *et al.*, 2019^(44)^	The food environment-quality index	University/ Technical and further education institutions	Inter-coder	–
Charoenbut *et al.*, 2018^(27)^	Questionnaire based on the CHEW and NEMS-S	Company	Cronbach’s alpha	Content validity
Horacek *et al.*, 2018^(41)^	A modified version of the NEMS-S	University	Interclass	–
Bortolot, Perez, Franco, 2019;^(42)^ Tavares *et al.*, 2021;^(50)^ Franco *et al.*, 2022;^(51)^ Batista *et al.*, 2023^(56)^	Instrument for analysing the university food environment	University	Inter-rater, test–retest	Content validity
Horacek *et al.*, 2019^(43)^	(VEND)ing audit	University	Inter-rater	Concurrent validity
Lee *et al.*, 2020^(45)^	NEMS-UC	University	Inter-rater, internal consistency	–
José, Castro, Canella, 2021^(68)^	Assessment Instrument of the Hospital Food Environment	Hospital	Inter-rater, intra-rater	–
Mann *et al.*, 2021^(46)^ Keat, Dharmayani, Mihrshahi, 2024^(47)^	The Uni-Food tool	University	Inter-rater	Face validity
Rodrigues *et al.*, 2021;^(49)^ Rodrigues *et al.*, 2023^(57)^	Questionnaire	University	Test–retest, internal consistency	–
Banerjee, Reddy, Gavaravarapu, 2022^(28)^	Checklist	Company	–	Content validity
Han *et al.*, 2022^(52)^	NESC-CC	University	Inter-rater, test–retest	–
Shokeen, Tamber, Sinha, 2022^(29)^	CHEW	Company	Intraclass	–
Quezada *et al.*, 2023^(30)^	NEMS-S	Company	Inter-rater	–
Li *et al.*, 2024^(58)^	C-NEMS-S	University	Inter-rater, intraclass	–

WEM, worksite environment measure; EAT, Environmental Assessment Tool; U-NEAT, The University Nutrition Environment Assessment Tool; U-NPAT, The University Nutrition Program Assessment Tool; HEATWAI, Healthy Trucking Work-settings Audit Instrument; CHESS, Community Health Environmental Scan Survey; NEMS-V, Nutrition Environment Measures Survey Vending; HNES, Hospital Nutrition Environment Scan for Cafeterias, Vending Machines, and Gift Shops; WEBS, Worksite and Energy Balance Survey; NEMS-R, Nutrition Environment Measures Survey for Restaurants; NEMS-CD, Nutrition Environment Measures Survey for campus dining; NEMS-S, Nutrition Environment Measures Survey for stores; CHEW, Checklist for Health Promotion Environments at Worksites; NEMS-V, Nutrition Environment Measures Survey Vending; NEMS-GG, Nutrition Environment Measures Survey Grab-and-Go tool; NEMS-UC, Nutrition Environment Measures Survey for University Campuses; NESC-CC, Nutrition Environment Scoring for Chinese Style University/Work-site Canteens; C-NEMS-S, Chinese Version of the Nutrition Environment Measurement Tool for Stores.

We synthesised the main limitations pointed out by the authors of the articles. The most frequent limitation was the lack of generalisation of the results, reported by the authors of eighteen articles (41·9 %), as samples were limited or non-representative in terms of geographical location or population; however, this does not refer to the instruments themselves. Another frequent limitation directly related to the instruments was the methods adopted for reliability analysis, referred to in 6 (14·0 %) studies. Problems related to self-reporting and memory bias were also mentioned (Table 3).

**Table 3:** Limitations pointed out by the authors concerning articles included in the present review (*n* 43), 2024

Limitations	Frequency	
	*n*	%
Generalisability of the results	18	41·9
Small sample size	8	18·6
Reliability methods	6	14·0
Inaccurate self-reported data	3	7·0
Cross-sectional design	2	4·7
Possible biased results	2	4·7
Recall bias	2	4·7
Sampling bias	2	4·7

## Discussion

The objective of this scoping review was to systematise the instruments available for evaluation of the organisational food environment of workplaces and their components and dimensions. In total, fifty-four articles were included and thirty-six instruments were identified. Of these, twenty-five were original instruments for evaluating the organisational food environment of workplaces, and eleven referred to variations or adaptations of NEMS versions. Regarding the evaluated places, nineteen instruments were used in universities, eight were used in hospitals and eleven were used in companies. Most studies used objective measures, and only eight studies used subjective measures. No instrument included all components and dimensions of the model in use. Of the thirty-six instruments, twenty-nine reported at least some measure of validity or reproducibility.

Almost half of the studies found in our review are from the United States. This result is similar to the one reported in a previous scoping review aimed at mapping instruments for the evaluation of obesogenic environments aimed at adults, which included studies published between 1997 and 2018^(7)^. This result is consistent with the fact that the United States is one of the pioneer countries in studies on food environments^(10)^. However, in our review, we also found a considerable volume of research conducted in Brazil, a country with an increasing number^(71)^ of relevant studies on the theme in Latin America^(72)^. As for the year of publication, we found studies published as early as 2007; however, the volume of publication increased as of 2010. Lytle and Sokol, in their update of the review of McKinnon and collaborators – which sought to identify studies that measured different types of food environments – found a higher number of studies as of 2007 (34 articles), with sixty-seven articles in 2012^(8,73)^.

Most of the identified instruments were used in universities, followed by companies and hospitals. In their review, Lytle and Sokol found 432 articles, and ‘workplaces’ was the category with the lowest number of studies (9 articles). This result is similar to the one reported previously by McKinnon and collaborators: eleven studies. The two studies, despite including workplaces, did not clearly define what was considered as a workplace, which may have been a limitation in the identification of studies that evaluated these places^(8,73)^.

Of the instruments identified in this study, eleven were variations or adaptations of NEMS. Although NEMS is a well-established instrument in the literature, with reliability reported by several studies^(74)^ – except for the Nutrition Environment Measures Survey–Campus Dining (NEMS-CD) and the NEMS-University Campuses (NEMS-UC), which are focused on commercial establishments of universities and based on NEMS-R – none of the other versions of NEMS is specific for evaluation of the organisational food environment.

NEMS-S is the most widely used instrument among the studies analysed^(30,36,39,41,58)^. This fact indicates that, although the studies sought to evaluate the organisational food environment, many of them focused on the evaluation of establishments and the availability of food and beverages in the target places. This limits a more comprehensive understanding of the organisational food environment as these places do not always allow on-site eating. On the other hand, instruments such as the Environmental Assessment Tool^(18–20,65)^, which were developed to assess the physical and social environmental support of the workplace for the prevention of obesity in the United States, provide a more complete overview of the interactions between workers and the food environment^(18)^. The Hospital Nutrition Environment Scan for Cafeterias, Vending Machines and Gift Shops^(61,62,64,69)^ also expands this perspective by gathering data on different hospital food services, while adapting to the hospital context and its specificities^(62)^. Finally, the Instrument for Analysis of the University Food Environment^(42,50,51,56)^, which follows the principles of the Dietary Guidelines for the Brazilian Population^(75)^ in the university food environment^(51)^, reflects an approach that integrates nutritional guidelines to the university food environment and goes beyond the mere availability of food by considering other aspects. Notably, the choice of the instrument directly influences the depth of the evaluation of the food environment, reinforcing the need for tools that capture more comprehensively the different dimensions of the organisational environment.

The presence of NEMS-S variations in nineteen instruments that evaluate universities, and which were identified in our study, suggests that the evaluations of this environment are still very focused on the availability of food and beverages in establishments. In a recent systematic review summarising the tools and methods used for assessing the health of university food environments, it was reported that of the thirty-six included studies, 58 % were institutional-level audits, 17 % examined individual-level perceptions and 25 % combined both types. Most institutional-level audits focussed on assessing one aspect of the food environment, such as restaurants and vending machines. For studies that examined various spaces within the campus environment (38 %), comprehensive assessments were limited and most studies had to employ a combination of assessment tools. NEMS was the most used tool in all studies^(76)^.

None of the instruments identified in our study includes all the components and dimensions present in the conceptual model of Castro and Canella^(6)^, which reflects the limitations of the current approaches to the evaluation of the organisational food environment. The predominant focus on the internal level of eating spaces and the institutional level, to the detriment of the surroundings and the decision level, shows that the instruments still do not comprehensively address the influences on the food environment. However, it should be noted that the assessment of the surroundings may not make sense in all contexts or locations; moreover, when necessary, other instruments can be used to complement the evaluation. The low rate of inclusion of the decision component – present in only 19·4 % of the instruments – reinforces the need to consider and include governance aspects and institutional policies that affect food supply.

Regarding the dimensions, the most included by the instruments was the availability of food and beverages in eating spaces, in all the evaluated settings. The dimensions least included in the instruments were ambience and acceptability. As proposed by Castro and Canella,^(6)^ although availability is central to the measurement of food environments, the metrics need to be improved to support actions aimed at enhancing the environments. The instrument that covers more components and dimensions is that of José, Castro and Canella (2021), from Brazil^(68)^ which did not include the acceptability dimension only. Although it was developed to evaluate the hospital food environment, the instrument looked further into the organisational food environment. However, its scope resulted in a very long instrument, which, in principle, does not produce a general score or scale, which may hinder its use and the systematisation of results^(68)^. The other two more comprehensive instruments regarding components and dimensions are Australia’s The Uni-food Tool^(46,47)^ and the United States’ (VEND)ing audit^(43)^, which address eleven and ten of the fifteen dimensions, respectively.

The validation and reproducibility of the instruments should also be mentioned. Although almost 81 % of the reported instruments have some measure of validity or reproducibility, the lack of standardisation in the metrics makes it difficult to compare the instruments. The review by Lytle and Sokol, when updating the review of McKinnon and collaborators, had already indicated an improvement in the reliability and validity evaluation of the instruments identified between 2007 and 2015, compared with the instruments published between 1990 and 2007^(8,73)^. However, the current scenario still poses similar challenges. In their review, Dahl *et al.* (2024) reported a lack of validated tools for specific use within the campus food environment. Of the thirty-six studies included, 47 % used a validated tool and 53 % used an adapted version of a validated tool, while 47 % described the reliability of the tool^(76)^. It is noteworthy that a central issue that has been reported in the literature and that has also been found in our study is that the numerous types of instruments being used indicate a lack of consensus in the evaluation of food environments^(8,73,76)^. In this sense, important efforts have been made at the International Network for Food and Obesity/Non-Communicable Diseases (NCDS) Research, Monitoring and Action Support (INFORMAS) to develop evaluation protocols that can be comparable between countries^(77,78)^. On the other hand, to some extent, it reflects different perspectives, paradigms or references. For example, in Brazil, the Dietary Guidelines for the Brazilian Population considers the extent and purpose of industrial processing of food to establish its recommendations and points out that ultra-processed foods should be avoided. In this context, versions of NEMS, which consider light or diet foods as healthy options, are not aligned with Brazilian recommendations.

Among the limitations reported by the authors of the articles included in the present review, the most frequent was the indication that the data are not generalisable, and particularly as regards the instruments, the evaluation of reliability. A scoping review that mapped scientific research on food environments in Brazil found similar limitations; in general, the main ones were the possibility of bias in data collection, the use of secondary data and the lack of a representative sample^(71)^. A systematic review that evaluated the food environment in Latin America also reported the lack of representativeness in the studies as a limitation^(72)^. This scenario emphasises the need to improve both evaluation instruments and sample representativeness to ensure a more accurate and comprehensive view of food environments.

In our study, the option to include places other than companies that are also part of the organisational food environment, such as hospitals and universities, made it possible to find other instruments that had not been identified in previous reviews. The option not to restrict the language and geographic location also favoured the expansion of the findings. In addition, to the best of our knowledge, this is the first specific review of instruments for evaluating the organisational food environment of workplaces. Although the scoping review does not intend to make a quality assessment of the studies, we synthesised the limitations reported by the authors of the included studies, which helps to put the quality of the instruments into perspective. Another limitation is that, despite our comprehensive approach, there is a persistent challenge of publication bias in scoping reviews; however, we did not include only methodological studies, which helps to minimise this limitation.

## Conclusion

This review identified instruments for evaluation of the organisational food environment that were designed for different workplaces. No instrument covered all components and dimensions; however, some included most of them, and most of the instruments underwent some reliability and reproducibility assessment. The differences between the instruments and the reproducibility and validity analyses impaired the comparability between the instruments and the results generated by them. The evaluation of the organisational food environment of workplaces is important for monitoring, planning interventions and formulating public policies for these places, thereby improving workers’ health. To this end, reliable and validated instruments are necessary to comprehensively evaluate this environment.
